# Agricultural, socioeconomic and environmental variables as risks for human verotoxigenic *Escherichia coli *(VTEC) infection in Finland

**DOI:** 10.1186/1471-2334-11-275

**Published:** 2011-10-18

**Authors:** Katri Jalava, Jukka Ollgren, Marjut Eklund, Anja Siitonen, Markku Kuusi

**Affiliations:** 1 Department of Infectious Disease Surveillance and Control, National Institute for Health and Welfare, Helsinki, Finland

## Abstract

**Background:**

Verotoxigenic *E. coli *(VTEC) is the cause of severe gastrointestinal infection especially among infants. Between 10 and 20 cases are reported annually to the National Infectious Disease Register (NIDR) in Finland. The aim of this study was to identify explanatory variables for VTEC infections reported to the NIDR in Finland between 1997 and 2006. We applied a hurdle model, applicable for a dataset with an excess of zeros.

**Methods:**

We enrolled 131 domestically acquired primary cases of VTEC between 1997 and 2006 from routine surveillance data. The isolated strains were characterized by virulence type, serogroup, phage type and pulsed-field gel electrophoresis. By applying a two-part Bayesian hurdle model to infectious disease surveillance data, we were able to create a model in which the covariates were associated with the probability for occurrence of the cases in the logistic regression part and the magnitude of covariate changes in the Poisson regression part if cases do occur. The model also included spatial correlations between neighbouring municipalities.

**Results:**

The average annual incidence rate was 4.8 cases per million inhabitants based on the cases as reported to the NIDR. Of the 131 cases, 74 VTEC O157 and 58 non-O157 strains were isolated (one person had dual infections). The number of bulls per human population and the proportion of the population with a higher education were associated with an increased occurrence and incidence of human VTEC infections in 70 (17%) of 416 of Finnish municipalities. In addition, the proportion of fresh water per area, the proportion of cultivated land per area and the proportion of low income households with children were associated with increased incidence of VTEC infections.

**Conclusions:**

With hurdle models we were able to distinguish between risk factors for the occurrence of the disease and the incidence of the disease for data characterised by an excess of zeros. The density of bulls and the proportion of the population with higher education were significant both for occurrence and incidence, while the proportion of fresh water, cultivated land, and the proportion of low income households with children were significant for the incidence of the disease.

## Background

Verotoxigenic *E. coli *(VTEC) is a highly infectious causative agent for severe gastrointestinal infection with haemorrhagic diarrhoea, which can lead to haemolytic uraemic syndrome (HUS) or thrombocytopaenic purpura (TTP) [1]. Most of the diagnosed VTEC infections in developed countries, including Finland, are of the serogroup O157, but an increasing number of cases are found with non-O157 serogroups O26, O111, O91, O103 and O145 [2,3]. The majority of VTEC infections occur among young children, predominantly during the summer months [2-4]. VTEC infection is rare in Finland, with only 10-20 cases reported annually to the National Infectious Disease Register (NIDR) [5].

Several risk factors for VTEC infections have been identified from outbreak data, modelling studies, and descriptive and analytical epidemiological studies. Cattle are considered the main reservoir for VTEC [6-8]. Living, visiting or working in close proximity to a farm with cattle or having a household contact working in such a farm have been found to be important risk factors [9-11]. Cattle density or VTEC-positive cattle density have been established as important explanatory variables for VTEC infections [4,12-16] or cases of HUS [16]. Also farm density per area of land [14] and animal manure application to soil are important [13]. Undercooked beef meat or meat products, water, vegetables and unpasteurized milk, ice cream, juice and cider can be sources of VTEC infections [17], and consumption of burgers and cold meat cuts are established risk factors [9,18]. Pathogenic strains of VTEC have been detected in minced beef meat, beef meat cubes and cuts, raw cattle, sheep and goat milk and sprouts [11]. Lake water or contaminated municipal water systems have caused outbreaks of VTEC O157 [17,19,20] and consumption of untreated surface water is a risk factor for non-O157 [18].

Poisson distribution based regression models are standard models for count data, including for infectious disease surveillance data [21]. However, if the data has an excess of zeros, the assumptions of the Poisson distributed models are no longer valid. Models based on negative binomial distribution may slightly correct for this problem, but when there is a severe inflation of zeros, more specific models are needed. Zero-inflated and hurdle models have been developed to overcome this problem [21,22]. With the use of a complementary log-log link function, the hurdle model reduces to a standard Poisson regression if the data is Poisson distributed [22]. These types of models have been applied recently for modelling sleeping sickness [23], cholera prevalence [24] and bacterial counts [25].

The aim of this study was to identify explanatory variables for VTEC infections reported to the NIDR in Finland between 1997 and 2006. VTEC infection is a rare disease in Finland and we used Poisson distribution based hurdle models with variable selection based on in-depth questionnaires and known risk factors. We chose the hurdle model based on its ability to handle an over-dispersion of zeros and applicability to scarce data. The incidence rate of VTEC infection at municipality level was used as an outcome variable and explanatory variables included various agricultural, socioeconomic and environmental factors. These explanatory variables also included several factors that have not been studied with VTEC infections previously. The null hypothesis that the populations in different municipalities in Finland are at the same risk of contracting VTEC infection was tested using a Poisson distribution based hurdle model.

## Methods

### Microbiological characterisation

The presence, isolation and biochemical identification of VTEC bacteria from stool cultures was done as previously described [7]. The isolates were O grouped as previously described [7] with the specific O antisera obtained from THL (National Institute for Health and Welfare), Statens Serum Institute (SSI, Denmark) and Oxoid (Oxoid, Basingstoke, UK). The isolates of the O157 strains were phage typed as previously described [2]. Chromosomal DNA of the isolates was characterized by the internationally standardized pulsed-field gel electrophoresis (PFGE) method [7].

### Definitions for the microbiologically confirmed VTEC cases

#### Domestic case

A case of VTEC infection with no history of foreign travel during the two weeks prior to onset of symptoms.

#### Index case

The VTEC case with "earliest onset of symptoms"- date or isolation date within a household or outbreak.

#### Household case

A VTEC case living in the same household with the index case.

#### Outbreak case

Two or more epidemiologically and microbiologically linked VTEC cases caused by strains of identical subtype and not of the same household.

#### Sporadic case

A VTEC case with no epidemiological or microbiological link to another VTEC case.

#### Primary case

Index or sporadic case.

### Surveillance of VTEC in Finland

For all cases reported to the NIDR since 1997, date of birth, sex, place of residence, the earliest reported date, sampling date, travel information, and death notification were reported. An in-depth interview as a part of the surveillance has been conducted since 1998 [2], with the questionnaire reformulated in 2003. The interviews were conducted either by THL or the local health district. The information and question types that differed between the questionnaires were either used in conjunction or information was combined from the two forms. However, in some cases data were insufficient for a full analysis of the in-depth questionnaires. The study material is part of routine enhanced surveillance carried out in Finland. The data presented does not include any individual data so that any person could be identified. The interviewed case patients have given informed consent for the surveillance. The data was accessed from the NIDR based on the justification on the infectious disease law (583/1986) and regulation (786/1986).

The information that was available from both forms or could be obtained by combining information included: Place of residence (postal code), attending school or day care and having VTEC positives in the same household. Clinical information included presence of diarrhoea, visible blood in the stool, HUS or TTP as a complication, admittance to a hospital, and death. Certain risk factors were asked in one or both forms, including living or visiting farms with domestic animals, consumption of raw or undercooked meat, unpasteurized milk or untreated water, and swimming in natural water.

### Modelling

As the infectious disease surveillance data is count data and there was an excess number of zeros in the data (mean number of cases 0.31 per municipality with variance of 1.2), we developed a Bayesian Poisson complementary-log log (clog log) hurdle model [22,26] with a spatial correlation factor for taking into account the neighbouring municipalities [27,28]. If a Poisson or negative binomial distribution based count model is applied to data with an excess of zeros without addressing these mixtures, the model can be strongly affected. The hurdle model was chosen due to its ability to handle the over-dispersion of zeros. In the zero-inflated models the count of zeros is mixed into two components, while in the hurdle models the zero and positive counts are modelled by separate processes. Zero-inflated models are designed to handle excessive zero counts, while hurdle models can also be adjusted for too few zeros. As the data were quite scarce and there was no need to distinguish between structural and sampling zeros, the hurdle model was chosen for the present study [21,22]. The hurdle model is composed of two parts, a binary model (logistic regression) generating positive counts versus zero counts, and a zero truncated Poisson model generating positive counts only. In a hurdle model, the data is considered as being generated by a process that generates positive counts only after clearing a zero barrier, or hurdle. Until the hurdle is cleared, the process generates a binary response and the covariates associate with the probability for occurrence of the cases in the logistic regression part. After the hurdle is cleared, the process generates only a positive counts response and the covariates estimate the magnitude for the incidence of VTEC cases. We used a Bayesian full-likelihood approach to modelling the data, taking into account also the missingness in the covariate data (using imputation and missingness indicators).

Domestic primary cases of VTEC were included in the analysis as outcome variables for the 10-year follow-up period. These were assigned with home municipality according to information on the in-depth questionnaires or NIDR information using a municipality division of 416 municipalities in Finland in 2007. The explanatory variables for modelling included various agricultural, socioeconomic and environmental factors. The results from the descriptive epidemiology of the present study and literature were used to identify potential variables to be included into the model. We performed a univariable analysis in the hurdle model with all potential explanatory variables and in the multivariable analysis we tested those variables that had p-values < 0.20 in the univariable analysis. Of the correlated variables, only the most significant ones and one per correlation group were included in the final model within each group, as marked in Additional file 1. Correlation coefficients within each group (1-5) were significant and > 0.80. To identify explanatory variables in the multivariable model, Gibbs' variable selection was used [29] with all explanatory variables as listed in Additional file 1. Those variables with a posterior inclusion probability of > 0.5 in the Gibbs' variable selection were included in the final model. The significant variables were tested with cross validation in the univariable analysis to identify influential values. For details of the model, see Additional file 2 and Additional file 3.

The statistical packages used included R (version 2.11.1), SPSS (version 18), Arcgis (version 9.3) (for data pre-processing), WinBUGS (version 1.4.3) and Stata (version 9.2). The hurdle models are described in the Additional file 2 and Additional file 3.

## Results

### Description of the cases

Between 1997 and 2006, 247 cases of microbiologically confirmed VTEC infections were reported to the NIDR in Finland. There was an average annual incidence rate of 4.8 (range 1.9-12.0) per million inhabitants and 25 (10-62) cases on average were identified annually. Of all cases, 46 cases (19%) had a history of foreign travel; these cases were excluded from further analyses. Outbreak cases accounted for 29 (12%) and household cases for 77 (31%) of all the cases, and of these, 36 domestic index cases were enrolled. Altogether 131 domestically acquired primary cases were enrolled. Of the 131 cases, 95 (73%) cases were sporadic.

Of all 247 cases, 249 strains were isolated from primary stool cultures of patients or their close contacts with VTEC infection. Two patients had a dual infection caused by two VTEC strains. Of the 131 domestically acquired primary cases enrolled in this study, 132 VTEC strains were isolated: 74 (56%) were serogroup O157 and 58 (44%) were of the non-O157 serogroup; one case had dual infections of both serogroups. Of the O157 strains, 60 (81%) typed to sorbitol negative and 14 (19%) to the sorbitol positive O157 group. Within the O157 group, the most predominant phage types (PT) were PT2, PT88 or its variant, PT8 and PT4. The genomic DNA of the strains gave 33 and 50 different PFGE patterns for the VTEC O157 and non-O157 strains, respectively. The non-O157 strains typed into 20 serogroups, the most common O groups being O103, O145 and O26.

Of the 131 primary cases, 51 (39%) were aged younger than 5 years and 23 (18%) were older than 40 years. Half (66, 50%) were male, with the gender distribution also being equal in the age groups (data not shown). Interviews were conducted for 83 (63%) cases. The postcode for the home address was known for 25 (30%) of the cases. Secondary household VTEC positives were reported in 24 cases (29%).

The symptoms were typical of VTEC infection: 74 (94%) had diarrhoea and 69 (90%) visible blood in the stools. HUS developed in 21 (28%) cases, and of these, 5 (7%) had TTP; in total, 61 (74%) were admitted to a hospital, and none of the cases died. Information on the reported risk factors was scarce: of the 37 cases aged 0-6 years, 8 (22%) attended day care. Living or visiting a farm with domestic animals was reported in 17 (22%) cases. Unpasteurized milk products were consumed in 16 (20%) cases, raw or undercooked meat was consumed in 17 (24%) cases. Consumption of untreated water was reported in 6 (17%) and swimming in natural water in 3 (8%) cases.

### Modelling of the data

**Figure 1:**
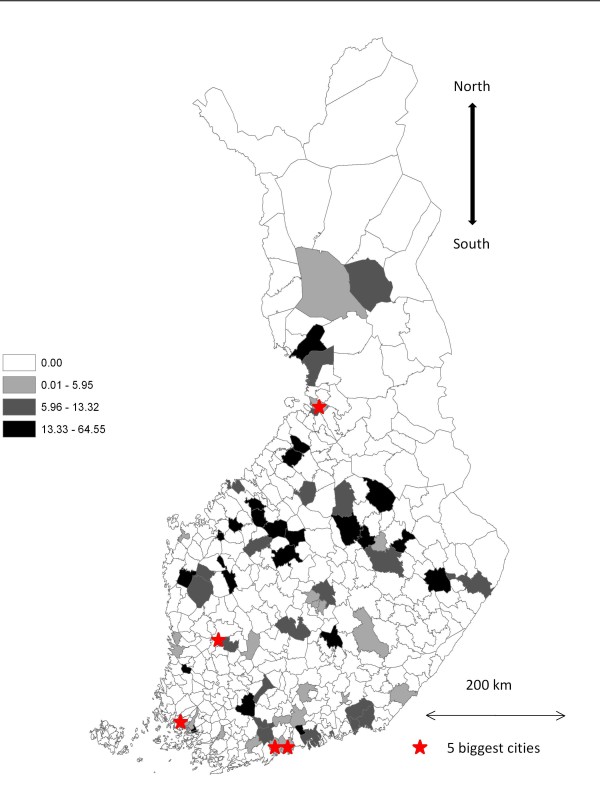
**Incidence rate of sporadic cases of VTEC infections per million inhabitants in Finland by municipality 1997-2006**.

The modelling unit was the municipality and between 1 and 11 cases were identified in 70/416 (17%) municipalities (Figure 1). Of all the cattle, socioeconomic and environmental variables, 17 variables were included in the multivariable models (Additional file 1).

In the multivariable model, bulls per human population and level of higher education per population were significant in the zero part of the hurdle models, indicating the importance of these variables for the occurrence of the infection (Table 1). The number of bulls per human population, the number of farms under cultivation per area, the proportion of fresh water to total surface area, the level of higher education per population and the proportion of low income households with children were positively significant in the zero-truncated Poisson of the hurdle models, indicating significance relating to the incidence of VTEC cases. The zero part of the hurdle model should be interpreted so that 1% change in bulls per population in a certain municipality or 1% change in level of higher education per population increases the approximated odds for the occurrence of VTEC by exp(0.01*β_bulls_/1.386)-1 = 4% and exp(0.01*β_educ_/1.386)-1 = 4%, respectively. Likewise, the 1% change in bulls, farms, fresh water, educational level and in low income households in the Poisson part increases the standardised incidence ratio (SIR) by exp(0.01*β_bulls_) = 5%, exp(0.01*β_farms_) = 2%, exp(0.01*β_fresh_water_) = 2%, exp(0.01*β_educ_) = 6%, exp(0.01*β_low_ income) _= 7%, respectively [30].

**Table 1:** Final multivariable hurdle models, with and without the spatial correlation variable

Variable (*)	Hurdle model with spatial correlation variable truncated Poisson	zero model	Hurdle model truncated Poisson	zero model
Bulls per population	5.0 (2.2-9.1)	5.0 (1.3-9.0)	5.1 (2.2-9.3)	4.8 (1.1-8.7)
Proportion of adult population with middle education or higher	5.6 (2.0-9.1)	5.0 (2.3-8.1)	5.8 (1.9-10.7)	4.6 (1.5-7.7)
Proportion of low income households with children	7.1 (1.9-12.4)	--(ns, excluded)	7.2 (1.9-12.3)	--(ns, excluded)
Number of farms under cultivation per square km	2.0 (0.7-3.2)	--(ns, excluded)	2.1 (0.5-3.3)	--(ns, excluded)
Fresh water per total surface area	1.9 (0.4-3.3)	--(ns, excluded)	1.9 (0.4-3.4)	--(ns, excluded)

(*) Parameter estimates (with 95% equal tail credible intervals)

Interestingly, using a cross validation procedure in the univariable or multivariable hurdle models, several more influential values were found. When two influential observations (in both zero and Poisson parts of the model) were removed from the data, the fresh water variable became negatively significant. When examining these municipalities in more detail, it became plausible that there may have been prolonged, undetected outbreaks. One of these municipalities was a medium size city with nine cases, with six cases during one calendar year and of those, four had identical PFGE profiles. The other was a small municipality with a population of just over 10 000 that had four cases during a two-year period with different PFGE patterns. Furthermore, these municipalities were among those with the highest proportion of fresh water, suggestive of water borne outbreaks. Also the number of cattle was high in these municipalities.

## Discussion

VTEC infection has a low average annual incidence rate of 4.8 per million inhabitants in Finland. The cases described in the present study were predominantly children with typical symptoms of VTEC infection: Most cases had haemorrhagic diarrhoea and some cases of HUS were identified. Half of the cases were infected with O157 serogroup strains. Detailed microbiological analysis including genotyping was essential to distinguish between outbreak and non-outbreak strains. Approximately a third of cases had a household contact who was VTEC infection positive, indicating direct transmission of the infection. Many of the cases reported visits to farms or consuming undercooked meat or unpasteurized milk; however, it was difficult to assess the impact of the risk factors obtained from the in-depth questionnaires as the information was scarce. Bias due to under-reporting of the cases is considered to be low for VTEC; however, diagnostic differences may occur between health care districts.

Hurdle modelling has not yet been widely used on infectious disease surveillance data [23,25,31]. One reason for using the hurdle model in the present study was the fact that the variance was much higher than the mean in the observed number of cases per municipality. The data was also characterised by an excess of zeros. As the zero and the truncated Poisson parts of the model gave different sets of significant variables, this further suggested the need for this type of model. If the coefficients of the binary part and the truncated Poisson part are close to each other, the hurdle model may be replaced by a standard Poisson regression model [22]. From the technical point of view, the prior distributions used were as weakly informative as possible, which guaranteed the convergence of the simulations. The results from the simulations were robust, especially to changes in the variances of the spatial factors. We also recommend using a full-likelihood approach in the multivariable analysis to explain the mechanism for the missing values.

We identified the proportion of beef cattle to human population and the proportion of population with higher education as being associated with increased occurrence and incidence of VTEC infections. The proportion of the population with higher education is likely to represent an indirect indicator of consumption habits, possibly eating undercooked beef steak. In addition, the number of farms under cultivation per area, fresh water per area and the proportion of low income households with children were associated with increased incidence of the infection. These are likely to represent routes for the spread of the infection; the low income households may be an indirect indicator of consumption habits. The model without the spatial variable essentially gave the same results. Our results concur with a US study on ecological factors for gastrointestinal infections, where the proportion of the population living on a farm (positive association), low education (negative association) and living in the south (negative association) were the most important socioeconomic factors for acquiring a VTEC infection [32].

The finding of beef cattle as a strong explanatory variable was not unexpected as cattle have been found to be an important risk factor for VTEC infections in epidemiological and modelling studies [9-11,13,14]. The VTEC prevalence is low in cattle in the Nordic countries, including Finland [33,34]. The overall cattle density [12] or dairy cattle or calves density [16] have also been found be significant, but these differences may reflect correlations between the cattle variables, variation in agricultural practices, and the ways in which data are collected. The significance of beef cattle over the other cattle variables may be due to a susceptibility of young animals to VTEC, raising the bull cattle in finishing units with multiple sources of animals, and the faecal contamination of the bulls and the environment during the finishing period [35]. The beef cattle density was an important factor both for the occurrence of the disease--confirming the role of cattle as a reservoir of the infection--and the incidence of the infection, indicating that contact with cattle may be an important route for spreading the infection. A relationship between human VTEC infections and VTEC positive cattle density has been found [14]. Also indirect cattle variables like the proportion of land where manure is applied has been found to be significant [13]; in the present study the proportion of cultivated land was also a significant risk factor for the incidence of the VTEC infections. Spreading animal manure on fields is common practice in Finland, with most of the manure coming from cattle [36].

The result for the proportion of fresh water surface per area is intriguing. Previous studies suggest drinking and swimming water as a source of VTEC infections and outbreaks [6,18-20,37]. However, two influential values had a strong effect for this variable in the present study. It would be interesting to examine the risk factors separately for water borne cases; this information was not widely available. There is recent evidence that contaminated water is an important route of infection in cattle [38] Campylobacter infections have been found to have a positive association with water pipe-length per population and a negative association with proportion of population receiving household water from a public supply [39]. It is noteworthy that the proportion of salted water was not a significant variable in the multivariable analysis. Finland has thousands of small lakes, but it also has a long sea coast along the western and southern borders. In a Swedish study, it was found that most of the VTEC cases lived along the coastline, near lakes or along rivers; however, the type of water was not specified [14]. VTEC prevalence studies have indicated that cases often have a private well or have experienced a water failure recently [12,40].

The finding that the proportion of the population with a higher education and the proportion of low income households with children being associated with increased incidence of the infection is somewhat contradictory. However, we feel that these two variables may explain different phenomena. There is conflicting evidence about educational level as a risk factor for VTEC infections [32,41]. However, it is known that people with higher education eat raw or undercooked ground beef more frequently [42], a known risk factor for VTEC infection [9]. The proportion of households where the main supporter of the household works in agriculture did not remain in the final model; however, there was a correlation between this and other agricultural variables. There may also be immunity: It has been found that in outbreaks, long-term exposure can lead to immunity [20]. In other studies, having a household member with an agricultural occupation was found to be a risk factor [9]. The most likely risk factor found for sporadic VTEC infection is having contact with a farm [11].

The study was limited by the low number of VTEC cases; therefore spatiotemporal analysis was not used. This was compensated for by using specific models designed for scarce data and performing a purely spatial analysis. The seasonal trends could not be evaluated properly due to the low number of cases. Another limitation of the study was the unavailability of data regarding consumption of beef or other food items per municipality. It has been found in Scotland that of all VTEC infections, 16% of cases had a significant food-related exposure, such as eating high-risk foods or having a contact working with meat [10]. Considering the VTEC infections in our study and the strong evidence of domestically acquired infections, transmission via imported food stuff would be plausible, based on the similar pheno- and genotypic characteristics of the non-sorbitol fermenting, sorbitol fermenting and non-O157 strains isolated in Finland and other European countries [7,43,44].

In conclusion, cattle are the main reservoir for VTEC infections as well as being also an important factor for the incidence of the disease. In addition, higher education per population was found to be associated both with the occurrence and the incidence of the disease, implying that food exposures may play a role. The role of fresh water and other environmentally related variables warrants further studies. Socioeconomic factors like low income and educational level expedite the incidence of VTEC infection. The recommendations for control measures for the prevention of VTEC infections given by the WHO include good hygiene in animal and slaughter process and food retail, consumer education on proper handling of foods, and up-to-date international and national legislation and regulation [45].

## Conclusions

We enrolled 131 cases of VTEC with 74 VTEC O157 and 58 non-O157 strains isolated (one person had dual infections). With Poisson hurdle models we were able to distinguish between risk factors for the occurrence of the disease (zero part of the model) and incidence of the disease (zero truncated Poisson part of the model) for data characterised by an excess of zeros in the case counts. The number of bulls per human population and the proportion of population with a higher education were identified as being associated with increased occurrence and incidence of human VTEC O157 and non-O157 infections. The proportion of fresh water per area, the proportion of cultivated land per area and the proportion of low income households with children were associated with increased incidence of VTEC infections. We recommend using a Bayesian framework with imputation for the missing values of the explanatory variables in conjunction with the missingness equations.

## Competing interests

The authors declare that they have no competing interests.

## Authors' contributions

KJ performed the analysis of the descriptive epidemiology data and the statistical analysis and drafted the manuscript. JO designed the statistical analysis and helped to perform it. ME performed the microbiological analysis and participated in the analysis of the descriptive epidemiology. AS participated in the design and coordination of the microbiological analysis. MK participated in the design and coordination of the epidemiological analysis. All authors read and approved the final manuscript.

## Pre-publication history

The pre-publication history for this paper can be accessed here:

http://www.biomedcentral.com/1471-2334/11/275/prepub

## Supplementary Material

Additional file 1 **Variables included in the modelling study and their sources**. List of agricultural, socioeconomic and environmental variables used in the present study. Click here for file

Additional file 2 **The statistical hurdle model for the VTEC infections**. The statistical hurdle model in detail used in the present study. Click here for file

Additional file 3 **Winbugs code for the hurdle model**. Winbugs code for the model used in the present study. This file can be viewed with Winbugs (http://www.mrc-bsu.cam.ac.uk/bugs/winbugs/contents.shtml) Click here for file
