# Supplementary material for: Agricultural, socioeconomic and environmental variables as risks for human verotoxigenic Escherichia coli (VTEC) infection in Finland
Source: BMC Infect Dis. 2011 Oct 18;11:275. doi: 10.1186/1471-2334-11-275 (PMC3226588; doi:10.1186/1471-2334-11-275)
Supplement: Additional file 1 — Variables included in the modelling study and their sources. List of agricultural, socioeconomic and environmental variables used in the present study. [file 1471-2334-11-275-S1.DOC]

Additional file 1. Variables included in the modelling study and their sources.

| **Variable** | **Source of the information** | **Data available for years** | **Mean value (s.d.)** | **Frequency (N=416, %)** | **Grouping (*)** | **Variables included in the final multivariable model (**)** |
| --- | --- | --- | --- | --- | --- | --- |
| Environmental variables |  |  |  |  |  |  |
| Summer cottages per square km (†) | Statistics Finland^2^ | 2006 | 2.9 (2.9) | (416, 100%) |  | * |
| Fresh water per square km | -“- | 2006 | 0.11 (0.15) | (416, 100%) |  | * |
| Salt water per square km | -“- | 2006 | 0.67 (2.48) | (416, 100%) |  | * |
| Agricultural variables |  |  |  |  |  |  |
| Cattle per population (Proportion of animals to number of humans) (‡) | Information Centre of the Ministry of Agriculture and Forestry in Finland^3^ | 1997-2006 | 0.55 (0.51) | (401, 96%) | 1  1 |  |
| Bulls per population | -“- | 1997-2006 | 0.06 (0.06) | (392, 94%) | 1 | * |
| Milking cows per population | -“- | 1997-2006 | 0.18 (0.18) | (389, 94%) | 1 |  |
| Calves per population | -“- | 1997-2006 | 0.18 (0.18) | (395, 95%) | 1 |  |
| Pigs per population | -“- | 1998-2006 | 1.01 (1.83) | (288, 69%) |  | * |
| Area of fields under cultivation per square km | -“- | 1997-2006 | 14.2 (11.4) | (413, 99%) |  |  |
| Number of farms under cultivation per square km | -“- | 1997-2006 | 0.46 (0.30) | (414, 100%) |  | * |
| 0-1/0-4/2-4/>5 year olds/all age groups (*) living in a farming household per population | Statistics Finland^2^ | 1995,2000,2004 | 0.078 (0.049) | (416, 100%) | 1 | * |
| Socioeconomic variables |  |  |  |  |  |  |
| Households per population | National Institute for Health and Welfare^4^ | 2000-2006 | 0.27 (0.014) | (415, 100%) |  | * |
| Proportion of children <18 years of low income households per  population <18 years | -“- | 2006 | 0.14 (0.049) | (410, 99%) |  | * |
| Households with four or more children  <18 years per population | -“- | 2000-2006 | 0.0073 (0.0045) | (416, 100%) |  | * |
| Households with three or more children  <7 years per population | -“- | 2000-2006 | 0.003 (0.0026) | (414, 100%) |  |  |
| Proportion of children of 0-1/0-3/0-6 (*) years attending public day care per population | -“- | 1997-2006 | 0.01(0.0085) | (415, 100%) | 2 | * |
| Proportion of children of 0-1(*)/0-3/0-6 years attending public child minding per population | -“- | 1997-2006 | 0.002 (0.00083) | (415, 100%) | 3 | * |
| Proportion of children of 0-1(*)/0-3(*)/0-6 years attending public day care and child minding per population | -“- | 1997-2006 | 0.0024 (0.0009) | (415, 100%) | 4 | * |
| Proportion of children aged between 14 and 16 not attending school lunch | -“- | 1997-2006 | 0.059 (0.028) | (289, 69%) |  | * |
| Average income per household | -“- | 1997-2005 | 4008.23 (3616.59) | (416, 100%) |  | * |
| Proportion of adult population with middle education or higher |  | 2006 | 0.56 (0.084) | (412, 99%) |  | * |
| Proportion of population with mother tongue other than Finnish or Swedish | -“- | 1997-2006 | 0.0099 (0.025) | (416, 100%) |  | * |

Footnote: (*) Of the correlated variables, the most significant ones were included in the model (**) Variables that were included in the multivariable models with a posterior inclusion probability of >0.50 in the Gibbs variable selection as described in the methods (†) Areas are referring to area of land by municipality (‡) Population data is referring to Finnish population by municipality

More detailed description of the variables used can be found at 1. [www.fmi.fi/en/](http://www.fmi.fi/en/), 2. [www.tilastokeskus.fi/index_en.html](http://www.tilastokeskus.fi/index_en.html), 3. [www.matilda.fi/servlet/page?_pageid=115,193&_dad=portal30&_schema=PORTAL30](http://www.matilda.fi/servlet/page?_pageid=115,193&_dad=portal30&_schema=PORTAL30), 4. uusi.sotkanet.fi/portal/page/portal/etusivu
