# Supplementary material for: Agricultural, socioeconomic and environmental variables as risks for human verotoxigenic Escherichia coli (VTEC) infection in Finland
Source: BMC Infect Dis. 2011 Oct 18;11:275. doi: 10.1186/1471-2334-11-275 (PMC3226588; doi:10.1186/1471-2334-11-275)
Supplement: Additional file 2 — The statistical hurdle model for the VTEC infections. The statistical hurdle model in detail used in the present study. [file 1471-2334-11-275-S2.PDF]

Additional file 2

The statistical model used in the present study is based on fitted random-effects Poisson complementary log-log hurdle model (25) allowing for spatial clustering (27). The hurdle model consists of two parts:

i) the probability π_0 that no human VTEC infections are detected in a given municipality
ii) probability of clearing the hurdle $\pi_+ = 1 - \pi_0$ in a given municipality and generating a non-zero count. Nonzero positive counts are distributed according to a truncated Poisson distribution.

Altogether the probability distribution of the observed cases of VTEC (Y) is:

$$Pr(Y = y) = \begin{cases} \pi_0 & \mathrm{y} = 0 \\ \dfrac{(1-\pi_0)e^{-\lambda}\lambda^y}{(1-e^{-\lambda})y!} & \mathrm{y} > 0 \end{cases}$$

The posterior distributions were calculated applying Gibbs sampling to likelihood function and as weakly informative priors as possible were used for unknown parameters and missing values. In the hurdle model the logarithm of parameters λ_i and π_{0i} were defined as follow:

$$\log(\lambda_\mathrm{i}) = \log(\mathrm{E_i}) + \alpha_0 + \sum_{\mathrm{k}=1}^{\mathrm{p}} \alpha_\mathrm{k} \times \mathrm{variable_{ik}} + \varepsilon_{\mathrm{i},1} + \mathrm{b_i}$$

$$\log(-\log)(\pi_{0\mathrm{i}}) = \log(\mathrm{E_i}) + \beta_0 + \sum_{\mathrm{k}=1}^{\mathrm{q}} \beta_\mathrm{k} \times \mathrm{variable_{ik}} + \varepsilon_{\mathrm{i},2} + \mathrm{c_i}$$

Here i represents the municipality $\mathrm{i} = 1, ..., \mathrm{N}$ (N= number of municipalities in Finland). The expected number of cases (E_i) was used as an offset in the regression model. It was calculated from the total number of VTEC infections (131) in humans in Finland between 1997 and 2006.The α_0 and β_0 are intercepts and α_k and β_k are variable coefficients.

The model was also adjusted for type of municipality with clustering effect, ε_1 and ε_2. The municipalites were classified to three groups according to the level of urbanisation (rural, population centre area and urban). To allow for spatial dependence (correlation) in the neighbouring areas, we assumed spatially correlated, independent random effects $\mathrm{b_i}$ and $\mathrm{c_i}$ (here $\mathrm{b_i}$ is used for either). The random effect $\mathrm{b_i}$ is a Gaussian variable which has the following distribution:

b_i=$(\mathrm{b}_1, ..., b_N) \sim \mathrm{MVN}(\mu, \mathrm{v}\Sigma)$

MVN is the N-dimensional multivariate normal distribution
μ is a mean vector with dimension 1 x N

v is to control for the overall variability of b_i s
Σ is a positive definite matrix with dimension NxN

We used the Besag's intrinsic version of CAR model (25) where the covariance matrix Σ is not positive definite as described in the Winbugs/Geobugs protocol (24).

Normally distributed noninformative priors were used, except for the spatial variable. Iterations were done until convergence was achieved, up to 200.000 iterations. The code for the Winbugs models is available (Additional file 3).
